# Miscellaneous Items

**Published:** 1886-05

**Authors:** 


### MISCELLANEOUS ITEMS.

**Our** next number will contain an article on the subject of *Mesmerism.*

**The Medical World.**—Philadelphia, states that onion tea, will often relieve sleepless and irriateable children, when opium and other narcoties have failed. Let the opium go, and try onions first.

**Peyrot**, of Paris, has made experiments in the transplantation of tendons. In a case where one of the popliteal tendons had been destroyed in a man, a fresh tendon was taken from a dog. This united and the function was preserved.

**Cure for Hiccough.**—According to the Lyons Medical, Dr. Grellety has observed that hiccough in children was immediately stopped by giving them a lump of sugar saturated with table vinegar. The same remedy was tried with adults with similar instantaneous success.

**Dr. John R. Featherstone.**—Who, for twenty years had been a practitioner of medicines in Indianapolis Ind., died from an overdose of Morpnine, which he had taken for Rheumatism with which he was much afflicted. One of many who die from a like cause but some of them do not know it.

**Captain** —What was the matter with your son when he died? "Nothing the matter with him when he died except that he was dead." "Pretty sick, though, before he died, wasn't he?" "Well he wasn't as peart as he had been." "Died a natural death, I suppose?" "Yes." "Whiskey?" "Whiskey."

**By** the use of cocaine, cancerous and other growths, may be painlessly removed. What a wonderful pain-killer it is, and yet its use is only just begun to be ascertained. It has been tried in seasickness and always failed of producing any good results. A drop or two of a two per cent. solution, will cure earache, if dropped into the ear.

**Bandaging** the legs with a wet cloth, and placing over that a layer of oiled silk, or oiled cotton is found to work like a charm in many cases of sleeplessness. The annoying sleeplessness of hot summer nights can usually be very readily relieved by using a rubber water bag, filled with cool water, as a pillow. The head is cooled, and sleep speedily makes itself felt.

**Priests** and Doctors used to be one, the priest assuming a devine eight to cure or kill people. When they did not know what to give to cure they betook themselves to prayer, incantation, amulets and performances of various religious ceremonies, the same as numerous scury tricks of the present day. When a man got well in spite of all their performances it was recorded as a cure. If he died the gods killed him. Now, if he dies, "his time had come."

**Childbirth Under Mesmerism.**—Dr. Edward Pritzel, of Vienna, reports the case (*Wiener Med Woch.*) of a young woman, aged twenty-six years, who entered the maternity hospital of Professor Braun. It was found that she was very susceptible to hypnotic influence, and when her labor came on, as it was slow and painful, she pas hypnotized. While in this state of artificial anesthesia the child was born, with no pain and with but slight loss of blood.

**Shall** medical quackery be suppressed? is the heading of an article in favor of making anything quackery that is not "regular." Ah! do you remember the history of the anti-slavery fight? shall abolition be suppressed? said some; yes, said the legislation of the nation; but it only made the friends of slavery mad and they thought they would fix their slavery forever. So they said but how? "Whom the gods intend to destroy they first make mad." Don't be too careful. Earn the first place and you will always retain it.

**Color Blindness** is a peculiar defect in the sight of some persons, first established as a fact by a chemist named Dalton, who labored under the deficiency. The remarkable infirmity sometimes exists in children for years before it is discovered, leading to misconceptions regarding the apparent stupidity of the sufferer. The sight is usually quite good in every other respect, but is more or less unable to perceive the differences or shade of colors, and in extreme cases there is no apparent difference to the defective eye between the color of the carnation and the green plant upon which it grows; red and green being the colors which appear to blend most thoroughly. In some instances the infirmity goes on undiscovered for years, because the sufferer is of course unaware of it, and if nothing remarkable occurs in respect thereto, others have no reason to suspect it. It is said that painful cases of this defect have occurred with reference to railway signals, where the sufferer has been unable to distinguish with certainty between a red or green signal, which has at length given rise to a strict investigation of applicants for positions, upon whose ability to distinguish colors depends the safety of trains and passengers.

